# Leishmaniasis in Uganda: Historical account and a review of the literature

**DOI:** 10.11604/pamj.2014.18.16.1661

**Published:** 2014-05-04

**Authors:** Joseph Olobo-Okao, Patrick Sagaki

**Affiliations:** 1 Department of Microbiology, College of Health Sciences, School of Biomedical Sciences, Makerere University, Kampala, Uganda; 2 Med Biotech Laboratories, Kampala, Uganda; 3 Amudat Hospital, Amudat district, Moroto, Uganda

**Keywords:** Leishmaniasis, Uganda, management

## Abstract

Visceral leishmaniasis (VL) or kala azar is a fatal and neglected disease caused by protozoan parasites. It occurs worldwide including north-eastern Uganda. This review gives a historical account of and reviews available literature on VL in Uganda to raise more awareness about the disease. Information was collected from: MEDLINE searches; records of Ministry of Health (Uganda), Amudat hospital records; records of NGOs and multilateral institutions; dissertations and personal communication. Results show that VL in Uganda was first reported in the 1950's, followed by almost four decades of neglect. Earlier records from the ministry of health and Amudat hospital on VL are also incomplete. From early 2000, reports mainly on the disease management and risk factors, started to appear in the literature. Management of VL has mainly been by NGOs and multilateral institutions including MSF Swiss. Currently DNDi is funding its management and clinical trials in Amudat hospital through LEAP. New cases of VL were reported recently from Moroto and Kotido districts and more patients continue to be received from these areas. In conclusion, management of VL is well established in Amudat hospital. However its sustainability and wider coverage remains a challenge. First-line drugs have now been registered in the country. Visceral leishmaniasis is apparently more widespread in north-eastern Uganda than originally thought. Research and surveillance on leishmaniasis is still weak. Strengthening the capacity of local institutions to; conduct surveillance and research, combined with effective management should mitigate VL in Uganda

## Introduction

Leishmaniasis is a disease complex caused by protozoan parasites belonging to the genus *Leishmania*. It was named after Leishman who first described it in 1903 [1]. Over 20 species of *Leishmania* can cause infection [2]. Leishmaniasis exhibits a wide spectrum of clinical manifestations which is dependent on the interplay between parasite and vector species, host's immune status and genetic factors [3]. The main forms of the disease are: simple cutaneous (caused by e.g. *L.major, L.tropica*); diffuse cutaneous (caused by e.g. *L. aethiopica*); mucocutaneous (caused by e.g. *L. braziliensis, L. panamensis*) and visceral leishmaniasis (VL) caused by parasites belonging to the *L.donovani* complex (*L. donovani and L.infantum*). Visceral leishmaniasis is the most severe form of the disease. Its characteristics include fever, weight loss, hepatosplenomegaly, anaemia and is fatal if not treated [3]. Post-kala-azar dermal leishmaniasis (PKDL) is a form of VL characterised by maculopapular or nodular lesions on the face, trunk or limbs, which normally occur following successful chemotherapy [4].

Parasites responsible for leishmaniases are transmitted by female sand flies following the bite for a blood meal. In the Old World and New World, sand flies of the genus *Phlebotomus* and *Lutzomyia* are known vectors of leishmaniases respectively. There are about 70 potential vectors but only 30 are proven vectors of leishmaniases [3, 5–7].

During feeding, promastigotes are deposited in the skin, where they are taken up by macrophages and neutrophils in which they transform into amastigotes. Promastigotes can also be cultivated *in vitro* and are elongated, flagellated and motile, while amastigotes are obligate intracellular and have no free flagellum, non-motile and oval in shape. The outcome of an infected sand fly bite to a human host ranges from no infection to asymptomatic infection to subclinical or to overt disease. Visceral leishmaniasis occurs in over 65 countries and has an annual incidence of 500,000 cases worldwide with about 200 million individuals at risk of contracting it [5]. More than 90% of global VL is concentrated in eastern Africa, Indian subcontinent and South America [5]. In Africa, the disease was earlier reported from Sudan, Ethiopia, Djibouti, Somalia, Kenya, Uganda, Chad, Niger, Gambia, Central African Republic and Gabon [8]. North Africa has a form of VL caused by *L.infantum* [9, 10] Eastern Africa has one of the world's highest concentrations of VL with endemic foci in Sudan, Ethiopia, Kenya, Uganda and Somalia [11].

The objective of this manuscript is to give a historical account of and review available literature on VL in Uganda with the aim to raise more awareness about the disease which is so highly neglected in the country.

## Methods

Information on VL was collected from: Amudat hospital records. The hospital is located in the VL endemic area and is the only health centre in Uganda with the capacity to treat VL. MEDLINE searches; records of Ministry of Health (Uganda), reports of NGOs and multilateral institutions; dissertations and personal communication.

## Current status of knowledge

### VL in Uganda

Karamoja region is a remote and semi arid area in north eastern Uganda. It is comprised of Moroto, Kotido, Kaabong, Abim, Nakapiripirit and Amudat districts. The region extends over 27,000 square kilometers with a population of about 1 million. The inhabitants of Karamoja are mainly semi nomadic pastoralists including the Pokot, Pian, Matheniko, Bokora, Tepeth, Jabwor and Jie. The Pokot tribesmen are found mainly in Amudat district while the other tribesmen inhabit the other districts. Most of the inhabitants of the region are poor and literacy rates are the lowest in the country [12]. The region experiences harsh climatic conditions with torrential rains and floods contrasted by high temperatures and drought and are generally marginalized and road network is poor and generally impassable during the rainy seasons [12].

Visceral leishmaniasis was initially reported in Uganda in two males from Karamoja region, north eastern Uganda in 1951 [13]. Four other cases from Karasuk were reported later in 1957 and treated in Kapenguria, Kenya [13]. A further two cases from Karamoja district were treated about the same time at Moroto hospital, Karamoja, Uganda [13]. More reports revealed that cases of kala azar were present in Karamoja, Uganda and could be confused with other liver diseases [14].

While cases of VL were reported initially at Moroto hospital [13, 14], no more subsequent reports were made from the same hospital. Instead, after a prolonged period, reports started to appear from Amudat hospital which is about 100km southwards [15]. Reasons for lack of continuity of treatment of VL at Moroto hospital are unclear.

Visceral leishmaniasis is now established to be endemic in certain foci in Pokot area, Amudat district, Karamoja region [16]. This belt extends into West, North and East Pokot, and Baringo districts in Kenya [17]. The Pokot people in Uganda know the disease locally as ‘Termes’ while the Pokot from Kenya call it ‘Noakta’. This is an indication that the local communities are familiar with the disease. This could be exploited for the control of the disease.

In Uganda VL is caused by *Leishmania donovani* parasites [18] and molecular studies to confirm this is ongoing under DNDi funding through LEAP to compare with isolates from Sudan, Ethiopia and Kenya. The clinical features of the disease are typical and include fever, weight loss, hepatosplenomegaly, anaemia [3]. Records of Amudat hospital indicated the prevalence to be higher among males than females and the disease peaks among the five to nine years and the ten to fourteen age groups [19]. Only few cases of PKDL have been observed in Uganda (Amudat hospital records. 2010) compared to the many cases reported especially from Sudan and to a lesser extent Kenya and Ethiopia [4].

### Vectors of VL

A limited study on vectors identified 14 species of sand flies in Amudat area, Karamoja region, north- eastern Uganda [20]. However, only *Phlebotomus martini* was found to be highly abundant in the area to bite man readily. *P. martini* had earlier been implicated as a vector of *L.donovani* in Kenya [21]. It was on the basis of the fly biting habits that it was incriminated as the vector for *L.donovani* in the Amudat area [20]. Hence more studies are needed on sand flies in Uganda. Transmission of VL can also be by other ways including mother to child transmission [22]. Recent hospital records of children born to VL positive mothers are being followed up for possible congenital transmission (Amudat hospital records).

### Amudat hospital

Amudat hospital is located in Amudat district. The district was recently created out of Nakapiripirit district. It is a rural mission hospital which was established in 1957 and has 120 beds about one third of which are for VL patients. It serves a population of about 100,000 people. It is the only hospital in Uganda today which has the expertise and facilities for the management of VL in the country. It is also a centre for clinical trials and biomedical research attracting both local and international collaborators [23]. Early records on management of VL in Amudat hospital are sketchy and missing (Amudat hospital records; 20,15). The records were presumed lost due to prevailing insecurity in the area then. However, available annual reports for 1983 and 1985 indicated VL as a frequent problem for admission in Amudat hospital [15]. A report by MSF, Swiss, showed a rising trend in the number of cases of VL in Amudat hospital between the year 2000 and 2005 [24]. It is however unclear whether the rise was due to increase in VL incidence or because of improved awareness, diagnosis, and reporting. But between 2005 and 2007 hospital records for VL cases were not reliable. This was the transition period between the MSF, Swiss team leaving and DNDi/LEAP taking over VL management at the hospital. However since 2008 to 2011 the number of cases of VL received in Amudat hospital has stabilised at about 80-200 per annum (Amudat hospital records). Patients at the hospital are mainly from Uganda with few from Kenya. This is contrary to an earlier report indicating that most VL patients at Amudat hospital were from Kenya [24]. A situation apparenlty reversed by opening another VL treatment centre, by MSF, at Kacheliba, in Kenya, about 60 km from Amudat.

### Extent of VL and reservoirs

**Figure 1:**
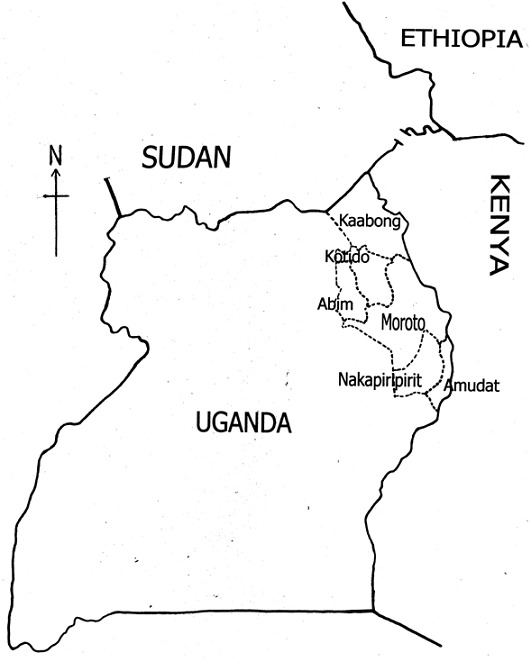
Map showing districts in north-eastern Uganda where cases of VL have been found

While knowledge on the extent of VL in Uganda is incomplete, a new focus for VL has been established outside the Pokot traditional focus, in Moroto district, about 100 km North of Amudat hospital (Amudat hospital records; Fig. 1). At the time of submission of this manuscript, other cases of VL were noted from Kotido district about 150 km from Moroto (Amudat hospital records). The Karamojong pastoralists from Moroto district know the disease with the local name of ‘Lokapet’. Patients from the areas denied any previous travel to the disease endemic foci in Pokot territory. Moreover, the majority of the cases were children below 12 years who were unlikely to have participated in any livestock raids in the known VL endemic areas (Amudat hospital records). This finding of VL outside Pokot area highlights VL as occurring also in other non Pokot communities in Karamoja region. It also confirms earlier reports of cases of VL from Moroto area [13]

The possibility of animal reservoirs for *L. donovani* parasites in Uganda has not been studied in detail. But in Kenya few isolates of leishmania parasites from dogs were reported [25]. One of the cases was from a dog in West Pokot, Kenya, an area adjacent to Pokot region in Uganda [25] (Figure 1). Since no new reports have appeared of more isolates from dogs from the region, we think that those were perhaps isolated cases. Hence VL in Uganda is likely an anthroponotic disease with humans being the sole reservoirs. Incidentally, cutaneous leishmaniasis is endemic in parts of Kenya [26, 27], Ethiopia [28] and Sudan [29], but only a single report of *L.aethiopica* in eastern Uganda was found in the literature [30].

### Risk Factors for VL

Risk factors for VL are malnutrition, immunosuppression due mainly to HIV infection and genetic susceptibility [31, 32]. Low socio economic status and treating livestock with insecticide are additional risk factors for VL in Karamoja region [33]. Moreover, sleeping near animals, owning a mosquito bed net and knowledge about VL were associated with reduced risks for contracting VL [33]. The main risk factors for in hospital deaths include age i.e below 6 years and above 15 years, concomitant tuberculosis or hepatopathy [34].

**Figure 2:**
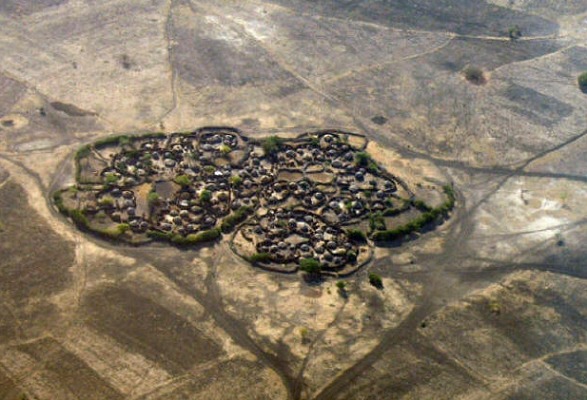
A manyatta near Moroto town, North East Uganda

**Figure 3:**
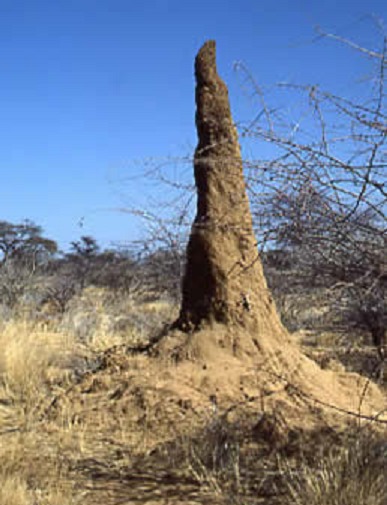
A Termite mound. *P. martini* sand flies are associated with termite mounds in VL endemic areas

The semi-nomadic lifestyle of the different communities in Karamoja sub region, coupled with limited pasture and water have created rivalries among them over the scarce resources and livestock which have resulted in intertribal conflicts and insecurity. Frequent movement of the population with their livestock in search of better pasture and water and population displacement due to inter tribal conflict, insecurity, famine and drought expose them to potential sand fly bites and possible infection with *Leishmania donovani* parasites. This is similar to situations in India, Sudan and Brazil [35, 36, 37]. Furthermore, although *P.martini* sand flies are associated with termite mounds in VL endemic areas, and most transmissions are thought to occur outdoor [38] (Figure 2, Figure 3), makeshift houses constructed from grass thatched roofs and walls made of mud and wattle could permit the flies to, easily enter, hide, and bite within the houses. More studies on local vector(s) for VL are needed to examine these potential risks (Figure 2, Figure 3).

### Laboratory Diagnosis of VL

Suspected VL patients reporting to the hospital for treatment are subjected to VL diagnostic algorithm which is currently under review (Amudat hospital records, MOH Uganda). In other cases, the community mobiliser moves within the community sensitizing them about VL. Suspected cases are then transported to the hospital for detailed examination and definite diagnosis of VL. Rapid diagnostic test kits based on rK39 antigen [39] have also been distributed in smaller health centres to be used as a screening test on suspected cases. Those found positive are transported to the hospital for further investigation. However, the dipstick can also be adapted as confirmatory test because of its high specificity and positive predictive value [39]. In addition to the rapid test kits [39] the direct agglutination test (DAT), [40] is also available locally, though it is more technologically demanding and requires well trained laboratory personnel to perform. Definite laboratory diagnosis involves taking spleen or bone marrow aspirates to demonstrate parasites in the tissues (Amudat hospital records).

### Treatment of VL

Chemotherapy for VL is currently undertaken only in Amudat hospital, Uganda which has the facilities and well trained and experienced personnel. First line drugs are the two pentavalent antimonial compounds, sodium stibogluconate (Pentostam^®^, SSG) or sodium antimony gluconate and meglumine antimoniate (Glucantime^®^) (Amudat) hospital records,^41^. The procurement of SSG and paromomycin (PM) in Uganda has been simplified by its registration in 2009 and 2011 respectively. Furthermore, the current multicentre clinical trials being conducted by LEAP with funding from DNDi [23] should provide better clues for more efficacious, safe and /or practical treatment regimen, in particular combination therapies [42]. Following recommendation by the WHO, a combination treatment using SSG and PM lasting 17 days instead of SSG alone for 30 days is at a pharmacovigilance stage at Amudat hospital [43]. Amphotericin B (Fungizone^®^) [41] and AmBisome are the second line drugs and are all available at Amudat hospital. All drugs used in management of VL patients in Uganda are provided by DNDi through the LEAP project.

### Control Strategies for VL

Globally, several strategies exist for the control of leishmaniasis. But overall, there is no recognized universal cost effective control package. The control strategy is generally dictated by the local disease conditions like the vector (s) and reservoir host(s). But case management based on accurate and early diagnosis and treatment is important in reducing morbidity and mortality [44]. This is the main control strategy for VL currently in Uganda (Amudat hospital records, MoH Uganda) Vector control through destruction of breeding sites or insecticide spraying has not been widely practised. Neither has personal protection using insecticide impregnated bed nets as for malaria [45]. Finally, better control strategies would be designed when studies to understand the biology of the vectors and animal reservoirs in the region are done.

## Discussion

Available reports on VL in Uganda first appeared in the 1950's [20]. This was followed by a period of nearly four decades of dearth. This indicated total neglect For VL which is due to several reasons. Visceral leishmaniasis is associated with poor communities in remote areas in developing countries [45, 46] and is considered a neglected disease worldwide [47, 48]. Karamoja is a hard to reach area. It has generally poor roads and limited road network. Harsh climatic conditions make the roads impassable in the rainy season as bridges could be washed off. Inter tribal rivalries brought about by limited natural resources like water, pastures and livestock and previous political instability and neglect have resulted in insecurity in the area [12]. The poor road network when combined with insecurity, though it has improved lately, makes travelling to and within the region difficult and risky.

Due to ignorance and illiteracy, and limited sensitization on availability of effective treatment for VL, most sick people seek local treatment from traditional doctors first. It is only after they have failed to heal that they seek alternative treatment from a health centre, generally when they are very sick and lucky enough to get there. Since there are few functional health centres, patients, most of whom are poor, have to walk long distances to get there. Public transport is generally unavailable, unreliable and expensive.

Leishmaniasis is one of the diseases that falls under Vector Control Division in the MoH, Uganda. The disease is under a general pool of diseases with no specific person assigned to handle it. Collection of epidemiological data on VL is irregular (MoH records, Uganda). The resource allocation for VL is either not there or too small as compared to other diseases like TB, HIV and malaria that pose bigger burdens to greater population in the country. Thus VL has been largely managed by NGOs and multilateral institutions including MSF (Swiss) and LEAP with funding from DND*i*. With little or no assistance from the government, VL is at stake especially when these institutions close their programmes. Furthermore, as a result of neglect by local and central governments and even academic institutions, there has been little interest in research resulting in no new knowledge in the general field of VL e.g vector biology, animal reservoirs and to a lesser extent treatment. Thus there is lack of local data to guide for a better approach for the disease control [49].

The finding of VL cases outside the traditional focus mainly from Moroto and to a small extent Kotido districts (Amudat hospital records) suggests that VL could be more widespread than originally supposed especially that VL is a non-notifiable disease and is found mainly in the rural semi-nomadic population [11]. Epidemiological studies should unravel the extent of the disease in the region. Efforts to build the capacity to control VL in Uganda and the region have been boosted by the creation of LEAP which has a current membership of Kenya, Sudan, Ethiopia and Uganda [23]. Current projects on clinical trials of new anti-leishmanial drugs and parasite speciation supported by DND*i* through LEAP should enable identification of better treatment options and understanding of the biology of the parasite in the region respectively. As a result of information obtained from a multicentre study conducted by LEAP partners including Uganda, and following the recommendation by WHO for treating VL using SSG and PM [43], a shorter course, combination therapy has been introduced in Uganda in a pharmacovigilance programme. The treatment lasts 17 instead of 30 days thus reducing the time and cost of treating VL [43].

## Conclusion

Management of VL in Uganda is well established especially in Amudat hospital. Diagnosis and treatment is the main control strategy. Although the dipstick meets many of the requirements, particularly in the remote peripheral health centres where laboratories are either absent or have ordinary equipment and the personnel are not so highly trained, better and more sensitive and specific diagnostic tests need to be developed [39]. Two anti-leishmanial drugs, sodium stibogluconate and paromomycin have now been registered in Uganda. Better and cheaper drugs would reduce the strain on the cost of treatment enabling more people to be treated especially that VL is a disease associated with poor communities in remote areas in resource limited countries [12, 45, 46]. Non-governmental organisations have been largely responsible for the management of VL including provision of drugs and diagnostic reagents because central and local governments do not provide adequate resources for its control. These authorities should thus take the centre stage to plan and provide funds for the control of VL and other neglected diseases to improve on the lives of their impoverished people. The cycle of neglected diseases which are associated with neglected systems and people would thus be checked. Visceral leishmaniasis is apparently more widespread in Uganda than previously estimated because new cases continue to be reported from Mototo and Kotido districts. Limited research and no surveillance have been conducted on leishmaniasis in Uganda. The research capacity needs to be strengthened and sustained to enable identification of the extent of the disease, better local research priorities and approaches to the disease control [49]. The current multi-centre clinical trials conducted by LEAP and funded by DND*i* is a clear example of needs driven research agenda [42]. The local communities in VL endemic areas know the disease well. Ownership of VL by the local people should therefore be intensified through community sensitization to achieve sustainable control strategies. Finally, insecurity, poverty, illiteracy and poor road network have all contributed to the neglect of VL. All these need to be addressed urgently and seriously, as they are grave impediments to the control of VL and other neglected diseases in Karamoja region and achievement of the MDGs target by 2015.
